# Variation within and between *Frankliniella* Thrips Species in Host Plant Utilization

**DOI:** 10.1673/031.011.0141

**Published:** 2011-04-06

**Authors:** Ignacio Baez, Stuart R. Reitz, Joseph E. Funderburk, Steve M. Olson

**Affiliations:** ^1^ USDA-ARS Center for Medical, Agricultural and Veterinary Entomology, 6383 Mahan Dr., Tallahassee, FL 32308; ^2^ North Florida Research and Education Center University of Florida, 155 Research Rd., Quincy, FL 32351; ^3^ Current address: USDA-APHIS, 1730 Varsity Dr., Suite 400, Raleigh, NC 27606

**Keywords:** fertilization, flower thrips, *Frankliniella occidentalis*, *Frankliniella tritici*, *Frankliniella bispinosa*, host selection, *Orius insidiosus*, pepper, tomato

## Abstract

Anthophilous flower thrips in the genus *Frankliniella* (Thysanoptera: Thripidae) exploit ephemeral plant resources and therefore must be capable of successfully locating appropriate hosts on a repeated basis, yet little is known of interspecific and intraspecific variation in responses to host plant type and nutritional quality. Field trials were conducted over two seasons to determine if the abundance of males and females of three common *Frankliniella* species, *F. occidentalis* (Pergande), *F. tritici* (Fitch) and *F. bispinosa* (Morgan), their larvae, and a key predator, *Orius insidiosus* (Say) (Hemiptera: Anthocoridae) were affected by host plant type and plant nutritional quality. Two host plants, pepper, *Capsicum annuum* L. (Solanales: Solanaceae) and tomato, *Solanum lycopersicum* L. that vary in suitability for these species were examined, and their nutritional quality was manipulated by applying three levels of nitrogen fertilization (101 kg/ha, 202 kg/ha, 404 kg/ha). *F. occidentalis* females were more abundant in pepper than in tomato, but males did not show a differential response. Both sexes of *F. tritici* and *F. bispinosa* were more abundant in tomato than in pepper. Larval thrips were more abundant in pepper than in tomato. Likewise, *O. insidiosus* females and nymphs were more abundant in pepper than in tomato. Only *F. occidentalis* females showed a distinct response to nitrogen fertilization, with abundance increasing with fertilization. These results show that host plant utilization patterns vary among *Frankliniella* spp. and should not be generalized from results of the intensively studied *F. occidentalis.* Given the different pest status of these species and their differential abundance in pepper and tomato, it is critical that scouting programs include species identifications for proper management.

## Introduction

Anthophilous flower thrips in the genus *Frankliniella* (Thysanoptera: Thripidae) exploit ephemeral plant resources and therefore must be capable of successfully locating appropriate hosts on a repeated basis. Host selection by such thrips is thought to be comprised of distinct stages. Orientation to and landing on a potential host plant (attraction), primarily based on visual cues, are followed by post-alighting testing to determine suitability for feeding and/or oviposition (acceptance) (Terry 1997). Plant type and nutritional quality are generally considered key determinants of host selection by polyphagous herbivores such as *Frankliniella* species.

Because of its global pest status (Reitz 2009), numerous studies have examined host colonization by western flower thrips *Frankliniella occidentalis* (Pergande). These studies have shown that across various agroecosystems, *F. occidentalis* will colonize many different hosts, but that definite differences or preferences do occur among available hosts in terms of adult or larval abundance (Yudin et al. 1988; Doederlein and Sites 1993; Pearsall 2000). In a laboratory study, Bautista and Mau (1994) found that *F. occidentalis* preference among five host plants changed as the plants went from the vegetative to flowering stage. Nitrogen is often considered the key nutrient required by herbivorous arthropods (Mattson 1980; Bernays and Chapman 1994), and feeding damage by *F. occidentalis* larvae has been positively correlated with aromatic amino acid concentrations among various accessions of each of four different crops (Mollema and Cole 1996). Several studies have found that *F. occidentalis* populations typically increase with increasing nitrogen fertilization of a particular host plant. Brodbeck et al. (2001) found that overall populations of adult *F. occidentalis* increased with soil fertilization of tomato, *Solanum lycopersicum* L, (Solanales: Solanaceae) and female abundance was most tightly correlated with increasing aromatic amino acid concentrations in flowers. Likewise, populations of adult and larval *F. occidentalis* increased with nitrogen fertilization of chrysanthemum, *Dendranthema grandiflora* (Tzelev) (Schuch et al. 1998; Davies et al. 2005). In contrast, Reitz (2002) did not find adult or larval abundance to be affected by nitrogen fertilization of tomato. Chen et al. (2004) found that *F. occidentalis* populations did not increase with nitrogen fertilization of *Impatiens walleriana* Hooker f, but did increase with increasing levels of phosphorus.

While these studies shed light on one particular thrips species, in the southeastern USA *F. occidentalis* is just one of several species of *Frankliniella* that commonly infest flowers of vegetable crops as well as numerous uncultivated plants (Chellemi et al. 1994). Two of the other common *Frankliniella* species inhabiting flowers are *F. tritici* (Fitch) and *F. bispinosa* (Morgan). Field surveys for these species across particular plants show that their abundance and distribution also vary interspecifically across plant species (Paini et al. 2007; Northfield et al. 2008). In contrast to *F. occidentalis*, densities of *F. bispinosa* and *F. tritici* adults do not appear to be related to nitrogen fertilization of tomato (Stavisky et al. 2002). These results suggest that these species do not utilize hosts in the same manner. Furthermore based on variations in sex ratios from field samples, it is likely that the sexes of each species do not utilize hosts in the same manner (Chamberlin et al. 1992; Reitz 2002). However, little is known of interspecific responses of these species to fertilization of hosts other than tomato.

While considerable information exists on the effect of plant type and nutritional quality on host selection by phytophagous insects, at least certain key pest species, less is known of how these factors affect predatory insects, especially zoophytophagous species. A key predator of *Frankliniella* spp. is *Orius insidiosus* (Say) (Hemiptera: Anthocoridae). Under field conditions, it can be quite abundant and prey heavily on thrips on plants such as pepper (Funderburk et al. 2000; Reitz et al. 2003), yet it is relatively uncommon in tomato (Pfannenstiel and Yeargan 1998). *Orius insidiosus* like other predatory Hemiptera feed on plants as well as prey, and plant species have significant effects on predator population dynamics (Coll 1996). Differences in how predators respond to variation in plant quality are not particularly well known, but limited data suggest the responses are variable. For example, abundance of *Orius* spp. did not differ with nitrogen fertilization of cotton, but abundance of other hemipteran predators such as *Geocoris* spp. increased with fertilization (Chen et al. 2008). The present study reports the host use patterns of these *Frankliniella* species across two plants species with varying nutritional quality, as manipulated by soil fertilization. Specifically, our goals were to determine 1) if different *Frankliniella* species show similar responses to different host plants; 2) if densities of thrips increased with nitrogen fertilization, 3) if females show a stronger response to host plant type and quality than do males; 4) if *O. insidiosus* densities are related to host plant type and quality. While previous studies have examined thrips distribution across either multiple types of hosts or within a single species treated with different fertilizer regimes, we are unaware of studies that have varied both host plant type and nutritional quality simultaneously. Field trials were conducted over two seasons when populations are characteristically high (in the spring) or low (in the fall) (Ramachandran et al. 2001; Reitz 2002) to determine if overall population abundance affects responses to host plants.

## Materials and Methods

Separate field studies were conducted at the North Florida Research and Education Center, Quincy, FL in the fall and spring. Tomatoes (‘Florida 91’ in the fall and ‘Florida 47’ in the spring) and pepper (*Capsicum annuum* L. ‘X3R Camelot’ in both fall and spring) were transplanted into plastic mulch covered beds on 4 August for the fall season and on 20 March for the spring season. In the fall season, beds were covered with white plastic mulch as a means to reduce soil temperature. In the spring season, beds were covered with black plastic mulch as a means to increase early season soil temperatures. Irrigation was provided by a drip tube underneath the plastic. Irrigation was based on evaporation measurements from the previous day. Beds were 90 cm wide and 180 cm apart from each other. There was one row of plants per bed. Tomatoes were staked and tied. Disease and weed management were the standards for northern Florida (Olson and Simmonne 2009). The only insecticide applied was *Bacillus thuringiensis* for control of Lepidoptera larvae.

Each experiment was arranged as a split plot design with whole plots arranged in a randomized complete block design. There were five replicate blocks used in each season. Three nitrogen fertilization treatments were assigned to whole plots within each block, and all fertilizer was applied at the time of bed formation (before transplanting). Each whole plot consisted of one raised bed, 24.4 m long and 0.9 m wide. The distance between whole plots within a bed was 12.2 m. The low rate of nitrogen fertilizer was 101 kg/ha of nitrogen, applied in a 6%N-8%P-8%K formulation. This amount and formulation was applied to all plots. Plots assigned to the medium rate of nitrogen fertilization received an additional 101 kg/ha of nitrogen in the form of ammonium nitrate (34% N), for a total of 202 kg/ha. Plots assigned to the high rate of nitrogen fertilization received an additional 303 kg/ha of nitrogen in the form of ammonium nitrate (34% N) for a total of 404 kg/ha. The low nitrogen rate represents one half of the recommended amount for north Florida; the medium rate is the recommended amount for north Florida, and the high rate is twice the recommended rate (Hochmuth 1997).

Split plots within each whole plot were the two plant types, pepper and tomato. Each split plot consisted of 20 consecutive tomato plants or 20 consecutive pepper plants. Plants were spaced 0.6 m apart within plots in each bed. Plants of each type were grouped into 5 sampling units of 4 plants each. Sampling was conducted every 3–4 days when plants were flowering, which lasted from May–June in the spring and from September–October in the fall. There were 7 samples days in the fall and 9 in the spring. On each sample date, five flowers from each of the five sampling units of plants per subplot were collected and placed in vials with 70% ethanol, for a total of 25 flowers per subplot per date.

All flowers were dissected, and samples were examined under a stereomicroscope at 50X. The species and sex of adult thrips were recorded. Because immature thrips cannot be identified to species, these were grouped together. *O. insidiosus* life stages and sex of adults were recorded. Representative voucher specimens are deposited at USDA-ARS, Tallahassee, FL.

Insect count data were expressed on a per flower basis for each sample. Data were analyzed separately for the spring and fall, by repeated measures analysis of variance (ANOVA) over time (Littell et al. 1991) by using Proc MIXED (SAS 2004). Nitrogen fertilization, plant type, and their interactions were included in the models. Because the three-way interactions did not improve model fit, these were omitted from the final models. An autoregressive covariance structure gave the best model fit for all dependent variables. The specific hypotheses of interest were if increasing nitrogen fertilization would result in increased abundance of thrips or their natural enemies; if there were differences between peppers and tomatoes in the abundance of thrips and their natural enemies, and if males and females of each species responded in a similar manner to host plants and fertilization regimes, and the variation in these responses over time. Additional regression analyses were conducted to determine if larval thrips populations were associated with the abundance of specific species of thrips or *O. insidiosus.* All data were logarithmically transformed before analysis to meet ANOVA assumptions.

## Results

Adults of *F. tritici, F. occidentalis*, and *F. bispinosa*, and larval thrips were substantially more abundant in the spring than in the fall, but responses to plant type and fertilization treatments were generally similar between seasons. In both seasons, *O. insidiosus* was the predominant species of all predators (>99.9%), but it was more abundant in the fall than in the spring.

### *Frankliniella occidentalis* populations

**Figure 1.**
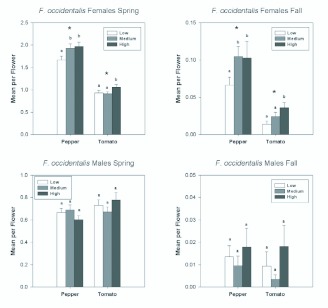
Overall seasonal mean numbers of female and male *Frankliniella occidentalis* per flower in tomato and pepper flowers grown under different nitrogen fertilization regimes during the spring and fall seasons. Data are means (+ SEM). Note different scale on y-axes for each season. Means for fertilization levels within each plant type marked with the same letter are not significantly different (P < 0.05, least squares means comparisons). Asterisks (*) denote significant differences in abundance between the two host plant types (P < 0.05). High quality figures are available online.

**Figure 2.**
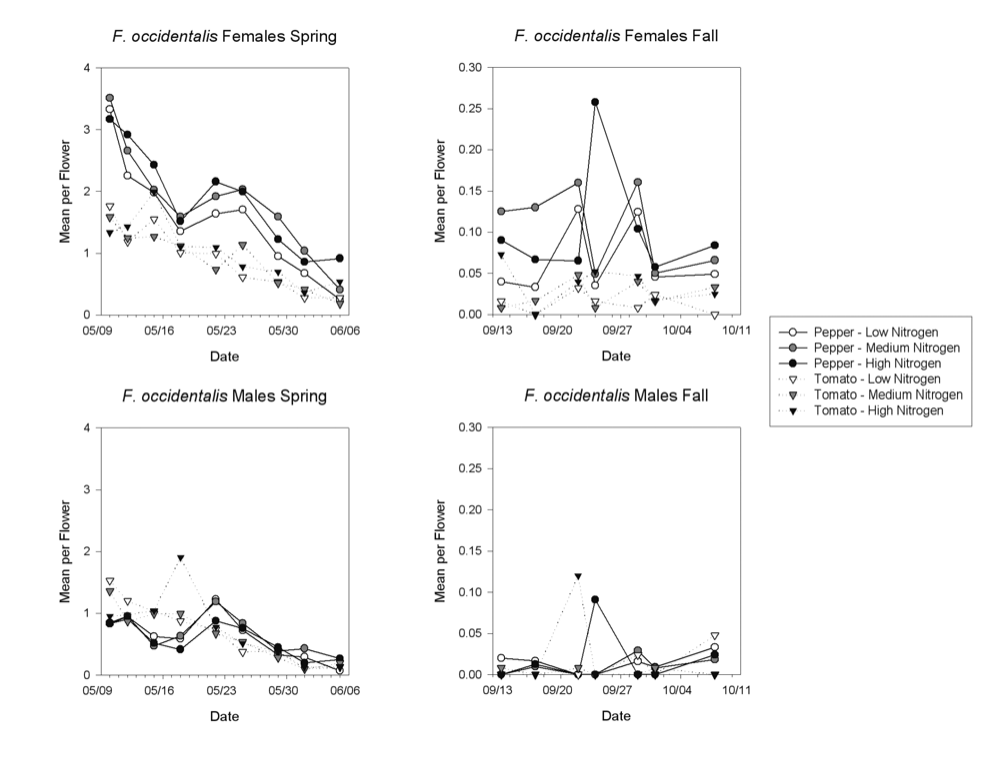
Mean densities of *Frankliniella occidentalis* females and males per flower in pepper and tomato grown under different fertilizer regimes on each sample date during the spring and fall seasons. Note the different y-axis scales between the spring and fall seasons. High quality figures are available online.

*F. occidentalis* was the most abundant thrips in pepper in the spring, where it comprised 64% of the adults. It comprised 22% of adults in tomato in the spring. In the fall, only 3% and 11% of adult thrips in pepper and tomato, respectively, were *F. occidentalis.* Females generally outnumbered males with mean sex ratios ranging from 73–90% female in pepper, in the spring and fall respectively, and 61– 84% female in tomato. Female *F. occidentalis* showed differences in abundance according to both plant type and quality (Figure 1). Female *F. occidentalis* were significantly more abundant in pepper flowers than in tomato flowers in the spring (Table 1; Figure 1) and in the fall (Table 2). In each season there was a significant date by plant type interactions, which resulted from the deceasing differences in abundance between pepper and tomato as each season progressed (Tables 1 and 2; Figure 2).

Female *F. occidentalis* were significantly more abundant (on a per flower basis) in the two highest nitrogen fertilization treatments for pepper in both the spring and fall (Figure 1). In tomato, there were significantly more female *F. occidentalis* in the highest nitrogen fertilization treatment compared with the medium and low treatments.

**Table 1.**
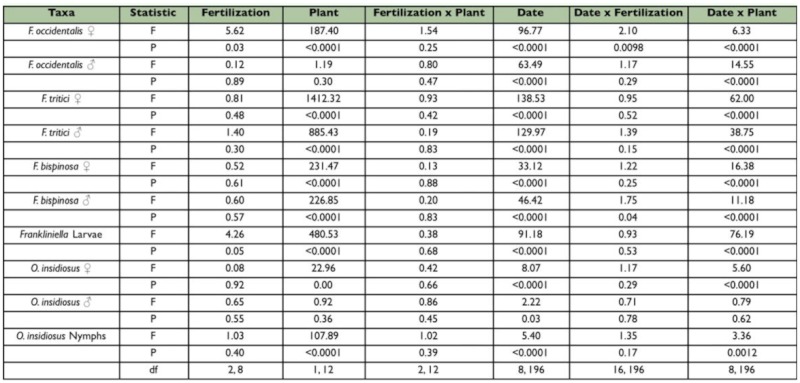
Analysis of variance results for the spring seasonfor the effects of nitrogen fertilization, plant type, date, and their interactions on the density of *Frankliniella* thrips and *Orius insidiosus* on a per-flower basis.No three way interactions were significant and therefore, were not included in the final models.

In contrast to females, *F. occidentalis* males did not show a differential response to plant type (Figure 1), as males were as abundant in tomato flowers as in pepper flowers in both spring and fall (Tables 1 and 2). Although there also were no overall differences in abundance of male *F. occidentalis* among fertilization treatments in the spring or fall (Figure 1), there was a significant fertilization by date interaction in both the spring and fall (Tables 1 and 2). These interactions resulted from relatively high numbers of males found in the high nitrogen treatments on certain sample dates (Figure 2).

### *Frankliniella tritici* populations

**Figure 3.**
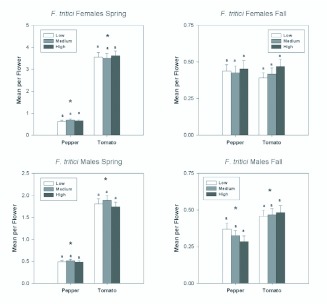
Overall seasonal mean numbers of female and male *Frankliniella tritici* per flower in tomato and pepper flowers grown under different nitrogen fertilization regimes during the spring and fall seasons. Data are means (+ SEM). Note different scale on y-axes for each season. Means for fertilization levels within each plant type marked with the same letter are not significantly different (P < 0.05, least squares means comparisons). Asterisks (*) denote significant differences in abundance between the two host plant types (P < 0.05). High quality figures are available online.

**Figure 4.**
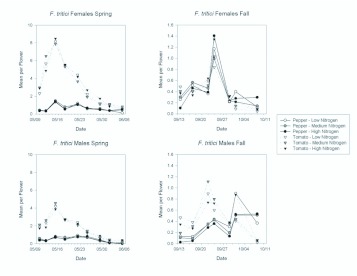
Mean densities of *Frankliniella tritici* females and males per flower in pepper and tomato grown under different fertilizer regimes on each sample date during the spring and fall seasons. Note the different y-axis scales between the spring and fall seasons. High quality figures are available online.

*F. tritici* was the most abundant thrips in tomato in the spring, when it comprised 68% of the adults, and in the fall when it comprised 78% of adults. In pepper, 30% of adult thrips in the spring and 81% in the fall were *F. tritici.* Sex ratios for *F. tritici* tended to be female biased, although less so than for *F. occidentalis.* In pepper, the sex ratios ranged from 56.5–66.6% female in the spring and fall, respectively. In tomato, sex ratios ranged from 57.7–48.8% female in the spring and fall. Fertilization treatments did not affect abundance of either *F. tritici* females or males in either the spring or fall (Tables 1 and 2; Figure 3). In the spring, *F. tritici* females and males were significantly more abundant in tomato than in pepper flowers (Tables 1 and 2; Figures 3 and 4). There was a significant date by plant interaction for both females and males in the spring because the difference in abundance between tomato and pepper declining later in the season. In the fall, females did not show a difference in host plant type (Table 2; Figure 3) although males did, with greater numbers again present in tomato than in pepper. There were significant date effects on male abundance indicating again the decreasing difference in abundance between tomato and pepper as the season progressed (Table 2; Figure 4).

**Table 2.**
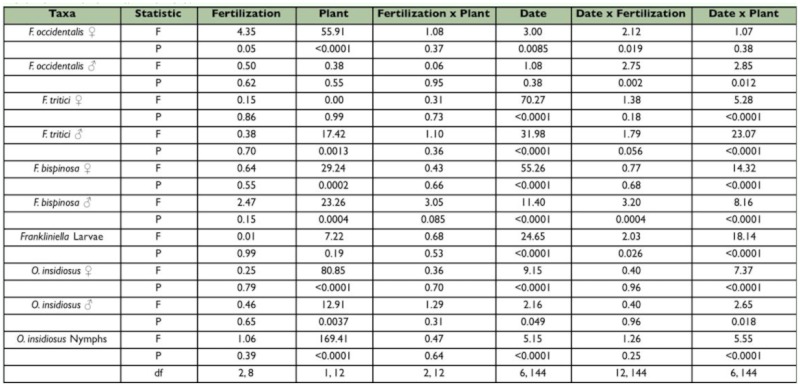
Analysis of variance results for the fall seasonfor the effects of nitrogen fertilization, plant type, date, and their interactions on the density of *Frankliniella* thrips and *Orius insidiosus* on a per-flower basis.No three way interactions were significant and therefore, were not included in the final models.

### *Frankliniella bispinosa* populations.

**Figure 5.**
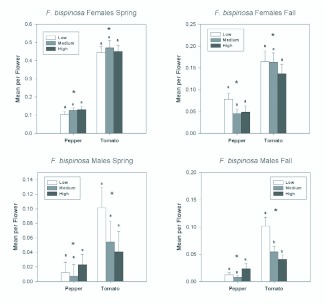
Overall seasonal mean numbers of female and male *Frankliniella bispinosa* per flower in tomato and pepper flowers grown under different nitrogen fertilization regimes during the spring and fall seasons. Data are means (+ SEM). Note different scale on y-axes for each season. Means for fertilization levels within each plant type marked with the same letter are not significantly different (P < 0.05, least squares means comparisons). Asterisks (*) denote significant differences in abundance between the two host plant types (P < 0.05). High quality figures are available online.

**Figure 6.**
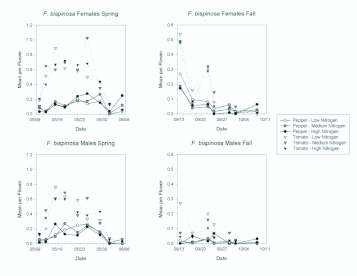
Mean densities of *Frankliniella bispinosa* females and males per flower in pepper and tomato grown under different fertilizer regimes on each sample date during the spring and fall seasons. Note the different y-axis scales between the spring and fall seasons. High quality figures are available online.

*F. bispinosa* tended to be the least abundant species. In the spring, it comprised 6% of adult thrips in pepper and 10% in tomato. In the fall, it comprised 8% of adult thrips in pepper and 19% in tomato. Sex ratios for *F. bispinosa* were nearly unbiased in the spring (50.4% female in pepper and 58.0% in tomato), but sex ratios were female biased in the fall (83.6% female in pepper and 70.3% in tomato). As with *F. tritici* females, fertilization treatments did not affect *F. bispinosa* female abundance in the spring or in the fall (Tables 1 and 2; Figure 5). However, *F. bispinosa* males were significantly more abundant in tomatoes at the lowest nitrogen treatment in the fall than in the other nitrogen treatments (Table 2; Figure 5). There were also significant date by fertilization interactions for male *F. bispinosa.* These resulted from day to day variation in differences in abundance among the treatments (Figure 6).

Similar to the results for *F. tritici, F. bispinosa* were significantly more abundant in tomato flowers than in pepper flowers (Figures 5 and 6). This difference held for females and males in the spring (Table 1; Figure 5), and in the fall (Table 2; Figure 5). There were significant date effects indicating changes in abundance over the seasons, and the interactions between date and treatment and plant resulted from the decreasing difference in abundances between tomato and pepper as the season progressed (Tables 1 and 2; Figure 6).

### Larval thrips populations

**Figure 7.**
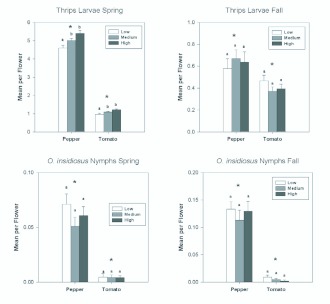
Overall seasonal mean numbers of larval *Frankliniella* thrips and *Orius insidiosus* nymphs per flower in tomato and pepper flowers grown under different nitrogen fertilization regimes during the spring and fall seasons. Data are means (+ SEM). Note different scale on y-axes for each season. Means for fertilization levels within each plant type marked with the same letter are not significantly different (P < 0.05, least squares means comparisons). Asterisks (*) denote significant differences in abundance between the two host plant types (P < 0.05). High quality figures are available online.

**Figure 8.**
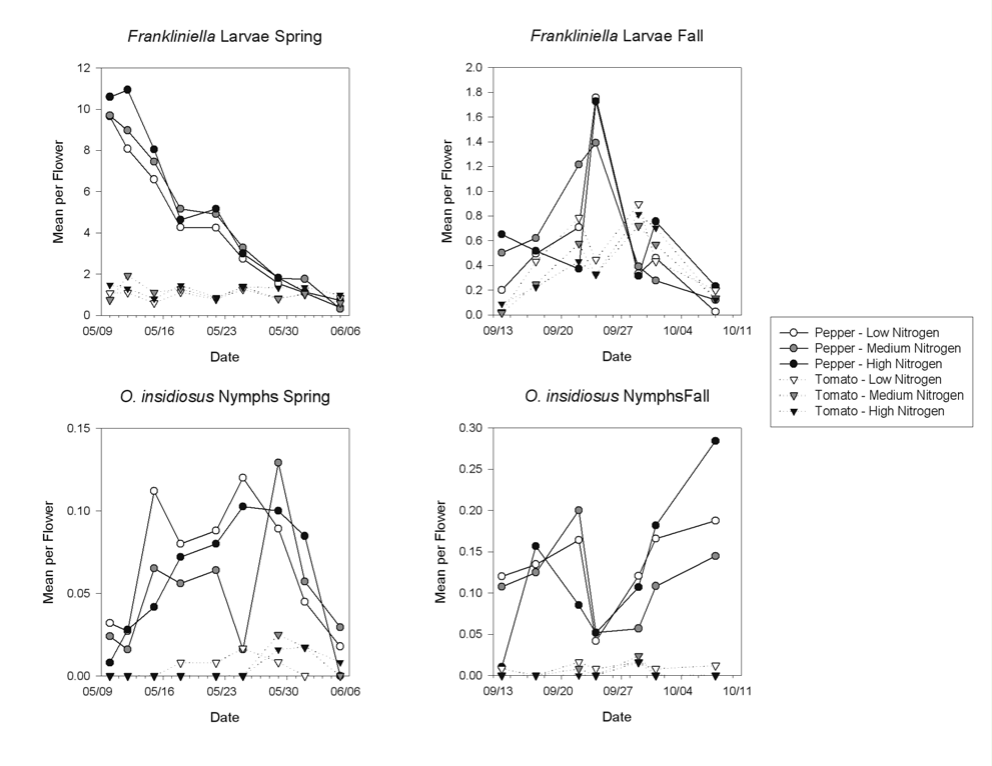
Mean densities of larval *Frankliniella* thrips and *Orius insidiosus* nymphs per flower in pepper and tomato grown under different fertilizer regimes on each sample date during the spring and fall seasons. Note the different y-axis scales among graphs. High quality figures are available online.

The abundance of thrips larvae followed a similar pattern to that of *F. occidentalis* females, being more abundant in pepper than in tomato. In the spring, there were almost 2.5 times as many larvae in pepper flowers as in tomato flowers (Table 1; Figure 7). In the fall, the difference was not as great but there were still significantly more larvae in pepper than in tomato flowers (Table 2; Figure 7). In the spring when larvae were more abundant in both pepper and tomato, there were significantly more larvae in the two highest nitrogen fertilization treatments than in the lowest treatment (Figure 7). There were significant date effects indicating changes in abundance over each of the seasons (Tables 1 and 2; Figure 8), but these interactions resulted largely from the substantial decreases in larval abundance in pepper flowers as the seasons progressed (Figure 8).

Multiple linear regression models showed that in the spring season, larval thrips populations in pepper were positively related to the abundance of *F. occidentalis* (test for coefficient = 0: t = 18.9, P < 0.0001) and *F. tritici* females (t = 4.64, P < 0.0001), but not to the abundance of *F. bispinosa* females (t = 2.32, P = 0.04). Larval thrips populations were negatively related to the abundance of *O. insidiosus* (test for coefficient = 0: t = -3.52, P = 0.004). The multiple linear regression model for the effect of these independent variables on larval thrips abundance in pepper was:

Log(larval abundance) = 0.207 + 1.01(log[*F.* *occidentalis* ♀]) + 0.329(log[*F. tritici* ♀]) - 0.19(log[*F. bispinosa* ♀]) - 0.427(log[*O.* *insidiosus*])

This model had an *R^2^* = 0.42. These same patterns were evident within each fertilization level except that *O. insidiosus* abundance had no effect on larval thrips abundance in the lowest nitrogen treatment. Larval populations in tomato in the spring were not significantly related to the abundance of females of any thrips species or the abundance of *O. insidiosus* (P > 0.5 for all parameter estimates).

In the fall season, the best fitting linear multiple regression model was:

Log(larval abundance) = 0.08 + 0.488(log[*F. tritici* ♀]) + 0.197(log[*O. insidiosus*])

This model showed that larval thrips populations in pepper were positively related to the abundance of *F. tritici* females (t = 0.8.73, P < 0.0001), and to the abundance of *O. insidiosus* (t = 2.18, P = 0.05), but this model had a low *R^2^* = 0.14. As in the spring, the same patterns were evident within each fertilization level for pepper except that *O. insidiosus* abundance had no effect on larval thrips abundance in the lowest nitrogen treatment. In tomato in the fall, larval thrips populations were related to the abundance of *F. tritici* females (P = 0.05), but this model:

Log(larval abundance) = 0.11 + 0.11(log[*F.* *tritici* ♀])

accounted for only 2% of the variation in larval abundance.

### *Orius insidiosus* populations

**Figure 9.**
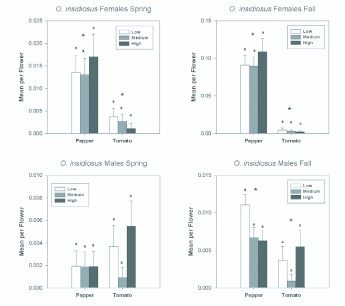
Overall seasonal mean numbers of females and males of *Orius insidiosus* per flower in tomato and pepper flowers grown under different nitrogen fertilization regimes during the spring and fall seasons. Data are means (+ SEM). Note different scale on y-axes for each season. Means for fertilization levels within each plant type marked with the same letter are not significantly different (P < 0.05, least squares means comparisons). Asterisks (*) denote significant differences in abundance between the two host plant types (P < 0.05). High quality figures are available online.

**Figure 10.**
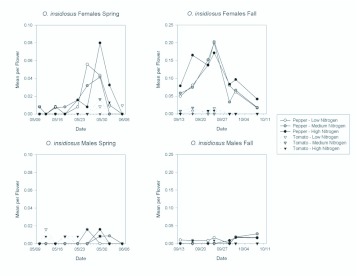
Mean densities of females and males of *Orius insidiosus* per flower in pepper and tomato grown under different fertilizer regimes on each sample date during the spring and fall seasons. Note the different y-axis scales among graphs. High quality figures are available online.

Even though thrips were less abundant in the fall than in the spring, populations of the key thrips predator, *O. insidiosus* were greater in the fall than in the spring (Figure 5). Sex ratios for *O. insidiosus* were exceedingly female biased in pepper, with a seasonal mean of 89.9% female in the spring and in the fall. In tomato, sex ratios were more variable between seasons. The mean sex ratio in tomato was 42.1% female in the spring and 80.0% in the fall. Females were 5 times as abundant in pepper as in tomato in the spring (Table 1 ; Figure 9), and 24 times as abundant in pepper as in tomato in the fall (Table 2; Figure 9). Female abundance changed over the course of each season (Tables 1 and 2), but there were generally significantly greater numbers in pepper than in tomato flowers (Figure 10). There was no effect of nitrogen fertilization on *O. insidiosus* female abundance in either season (Tables 1 and 2).

Few *O. insidiosus* males were found in the spring (Figure 9). Consequently, fertilization treatment and plant type did not appear to significantly affect their populations (Table 1 ; Figure 9). Male *O. insidiosus* were more abundant in the fall than in the spring, with significantly more being found in pepper than in tomato (Table 2; Figures 9 and 10). The significant date by plant interaction resulted from increasing greater abundance in pepper than in tomato as the season progressed. However, as in the spring season, fertilization treatment did not show an effect on *O. insidiosus* male abundance in the fall (Table 2).

Nymphs of *O. insidiosus* showed a strong response to plant type, with significantly greater numbers occurring in pepper than in tomato in both the spring and fall (Tables 1 and 2; Figure 7). There were significant date effects indicating changes in abundance over the seasons (Tables 1 and 2; Figure 8), but interactions between date and plant were consistent, with consistently greater abundances in pepper than in tomato.

## Discussion

Although these *Frankliniella* species are considered highly polyphagous (Chellemi et al. 1994; Paini et al. 2007; Northfield et al. 2008), it is important to note that both interspecific and intraspecific differences were found in host utilization by adults of these *Frankliniella* species. The differences among host plants found in the present study indicate these species are capable of active host plant selection (see Lewis 1997) and perhaps a degree of directed movement to particular hosts. All three species of these thrips are attracted to the white and yellow colors of pepper and tomato flowers (Walker 1974; Matteson and Terry 1992). If these species are highly attracted to both colors and the relative attractiveness is similar for both hosts, the ultimate host use decisions may be based on post-alighting cues.

Substantial populations occurred on all available hosts; therefore it appears that these species have preferences for particular hosts but not strict specificity. Female *F. occidentalis* showed distinct preference for pepper over tomato, whereas *F. tritici* and *F. bispinosa* females preferred tomato to pepper. Male *F. occidentalis* did not show a preference between pepper and tomato flowers. This may be associated with the generally low abundance of male *F. occidentalis*, as indicated by the highly female biased sex ratios in both seasons. In contrast, sex ratios for *F. tritici* and *F. bispinosa* were somewhat less female biased, and their males showed similar host preferences as females. However, these results are more likely to reflect fundamental behavioral differences between the sexes and among the species.

Male *F. occidentalis* can disperse more rapidly over distance than females (Rhainds and Shipp 2004). Males may be more likely to disperse and colonize hosts that favor mate location whereas females are more likely to select hosts that optimize oviposition and reproductive success (Terry and Kelly 1993; Rhainds et al. 2005).

Based on the abundance of thrips larvae and the ratios of larvae to females in pepper compared with tomato, it appears that pepper is inherently a much more suitable reproductive host than tomato. Therefore, the greater abundance of *F. tritici* and *F. bispinosa* females in tomato runs counter to expectations and to the results for *F. occidentalis* females. In addition, previous research has generally shown a positive relationship between host plant nitrogen content and abundance of *F. occidentalis* and other phytophagous insects (Slansky and Scriber 1985; Brodbeck et al. 2001; Chau et al. 2005). Our results showing greater abundance of *F. occidentalis* females in tomato with increasing nitrogen fertilization for *F. occidentalis* females are in agreement with these previous studies, with the exception of an earlier study of thrips abundance in tomato (Reitz 2002). This discrepancy may be attributable to the fertilizer applications in that study being made over time, which may have influenced plant responses differently from the single pre-plant application used in the present study. Abundance of *F. occidentalis* females in pepper showed similar patterns according to fertilization as in tomato; we are unaware of other studies examining *Frankliniella* spp. populations in pepper in response to fertilization. While fertilization effects on abundance of *F. occidentalis* were less pronounced than effects of plant species, fertilization effects were significant. Females would be expected to select hosts with greater nitrogen content as dietary nitrogen is associated with female fecundity and offspring performance (Trichilo and Leigh 1988). However, results for females of *F. tritici* and *F. bispinosa* ran counter to these expectations. Consequently, the decisions governing selection and acceptance of hosts appear to differ from those of *F. occidentalis.* It is possible there was an undetected fertilization effect on thrips abundance on a per plant basis. Flower number typically increases in a curvilinear manner in response to nitrogen fertilization, with numbers greatly increasing as nitrogen rates increase from suboptimal to optimal levels, but with little increase from optimal to supraoptimal levels (Johnson and Decoteau 1996; Brodbeck et al. 2001; Kang et al. 2004; Reitz SR, unpublished).

As reported in other studies (Funderburk et al. 2000; Reitz et al. 2003), pepper was found to be an excellent host for *O. insidiosus.* This predator moves rapidly through the environment and can quickly colonize pepper (Ramachandran et al. 2001; Reitz et al. 2003). *F. tritici* and *F. bispinosa* tend to be more active than *F. occidentalis* (Reitz et al. 2002; Reitz et al. 2006) and move throughout the environment more rapidly (Ramachandran et al. 2001). This inherent activity may be a predator avoidance tactic as both *F. tritici* and *F. bispinosa* are better able to avoid predation by *O. insidiosus* (Baez et al. 2004; Reitz et al. 2006), and they may preferentially move to plants like tomato to avoid *O. insidiosus.* In turn, these species may be better able to exploit other alternate host plants for reproduction (Paini et al. 2007).

*F. occidentalis* is the most important thrips pest globally and has been used as a model for understanding the biology and ecology of Thripidae. Our results demonstrate that congeneric *Frankliniella* species differ in the host use patterns and that male and females of these thrips can show differential responses to host plants and host plant quality. Furthermore, the findings that *F. tritici* and *F. bispinosa* have opposite patterns of abundance from *F. occidentalis* in two important crop hosts have important implications for pest management programs. *F. occidentalis* is a much more damaging species than either *F. bispinosa* or *F. tritici* (Funderburk 2009). Consequently, proper species identification is critical for the success of scouting programs for tomato and pepper.
